# Altered Fecal Microbiota Composition in Older Adults With Frailty

**DOI:** 10.3389/fcimb.2021.696186

**Published:** 2021-08-17

**Authors:** YuShuang Xu, YiHua Wang, HeWei Li, Yong Dai, Di Chen, MengMeng Wang, Xin Jiang, ZaoZao Huang, HongLu Yu, JuanJuan Huang, ZhiFan Xiong

**Affiliations:** ^1^Division of Gastroenterology, Liyuan Hospital, Tongji Medical College, Huazhong University of Science and Technology, Wuhan, China; ^2^Institute of Geriatric Medicine, Liyuan Hospital, Tongji Medical College, Huazhong University of Science and Technology, Wuhan, China; ^3^School of Mathematics, Shandong University, Jinan, China; ^4^Yangchunhu Community Hospital, Liyuan Hospital, Tongji Medical College, Huazhong University of Science and Technology, Wuhan, China; ^5^Liyuan Community Health Service Center of HongShan District, Wuhan, China

**Keywords:** frailty, gut microbiota, 16S rRNA sequencing, intestinal permeability, inflammation

## Abstract

**Objective:**

Frailty is a common geriatric syndrome that is diagnosed and staged based mainly on symptoms. We aimed to evaluate frailty-related alterations of the intestinal permeability and profile fecal microbiota of healthy and frail older adults to identify microbial biomarkers of this syndrome.

**Methods:**

We collected serum and fecal samples from 94 community-dwelling older adults, along with anthropometric, medical, mental health, and lifestyle data. Serum inflammatory cytokines IL-6 and HGMB1 and the intestinal permeability biomarker zonulin were measured using enzyme-linked immunosorbent assays. The 16S rRNA amplicon sequencing method was performed to determine the fecal composition of fecal microbiota. We analyzed the diversity and composition differences of the gut microbiota in the two groups and assessed the relationship between the changes in microbiota structure and clinical biomarkers.

**Results:**

Older adults with frailty showed higher concentrations of IL-6, HGMB1, and zonulin. Although there were no statistically significant differences in the diversity index and evenness indices or species richness of fecal microbiota between the two groups, we found significant microbiota structure differences. Compared with the control group, fecal samples from the frail group had higher levels of *Akkermansia*, *Parabacteroides*, and *Klebsiella* and lower levels of the commensal genera *Faecalibacterium*, *Prevotella*, *Roseburia*, *Megamonas*, and *Blautia.* Spearman’s correlation analysis showed that the intergenus interactions were more common in healthy controls than older adults with frailty. *Escherichia*/*Shigella*, *Pyramidobacter*, *Alistipes*, and *Akkermansia* were positively correlated with IL-6, while *Faecalibacterium*, *Prevotella*, and *Roseburia* were negatively correlated with IL-6. *Alistipes* were found to be positively correlated with HGMB1. *Akkermansia* and *Alistipes* were linked to the increased serum level of inflammatory factors and intestinal permeability.

**Conclusions:**

Frailty is associated with differences in the composition of fecal microbiota. These findings might aid in the development of probiotics or microbial-based therapies for frailty.

## Introduction

Frailty is a common geriatric syndrome with multiple causes and is characterized by increased vulnerability and functional impairment that decreases the ability to deal with stress (Morley et al., 2013). This health status mainly includes the loss of muscle mass and strength, reduced endurance, decreased physiological reserves, and cognitive impairment (Morley et al., 2013; Wilson et al., 2017; Pansarasa et al., 2019). Older adults with frailty are more prone to experience a series of clinical adverse events such as falls, disability, depression, institutionalization, and mortality (Pansarasa et al., 2019). Frailty is associated with an increased demand for long-term care and high medical costs (Hoogendijk et al., 2019). The prevalence rate of frailty among older adults in the community is 4.0%–59.1%, increases with age, and affects women more commonly than men (Collard et al., 2012).

Age-related changes to the immune system, including immune senescence and “inflammaging,” are thought to play a role in the frailty and sarcopenia process (Wilson et al., 2017). Levels of circulating inflammatory mediators increase with age, and the primary cause of this age-related inflammation may be related to microbial dysbiosis. Animal experiments from Thevaranjan et al. had shown that age-associated microbial dysbiosis promotes intestinal permeability and systemic inflammation (Thevaranjan et al., 2017). The gut microbiota, which is also referred to as the “the second genome” influences the human health status and has characteristics of adaptability and plasticity (Kim and Jazwinski, 2018; Durack and Lynch, 2019). Machine learning algorithms can be used to establish microbiome profiles that may predict the age of healthy individuals, indicating that gut microbiota may serve as a marker for aging-related research (Galkin et al., 2018). However, there are relatively few studies on the relationship between intestinal dysbacteriosis and frailty.

Here, we analyzed the serum levels of the inflammatory cytokines IL-6 and HGMB1 and the intestinal permeability biomarker zonulin in older adults living in communities. We characterized and compared the microbial community composition between frail older adults and healthy controls using 16S rRNA gene sequencing and analyzed the relationships between fecal microbiota and clinical characteristics.

## Methods

### Recruitment of Participants and Inclusion/Exclusion Criteria

Community-dwelling adults living in communities older than 70 were recruited and clinically investigated. All participants had been living in the community for at least 5 years and all of them belong to the yellow race. Older adults were confirmed to be suffering from frailty by applying a version of the Fried et al. definition for frailty (Fried et al., 2001). Exclusion criteria were malignant disease or advanced organic diseases, such as serious metabolic, cardiologic, hepatic, renal, and respiratory dysfunction; gastrointestinal diseases with definite diagnoses, such as inflammatory bowel disease, intestinal obstruction, and colon cancer; a history of abdominal surgery; receiving medications affecting intestinal microbiota, such as antibiotics or antipsychotic treatment within the last 30 days; or transformation of dietary structure, such as supplementation of probiotic preparation within the past 3 months. Age- and sex-matched older adults receiving a periodic physical examination were chosen as the control group. Healthy controls did not have major gastrointestinal inflammatory diseases such as inflammatory bowel disease. The collected information included anthropometric, medical, mental health, and lifestyle data, including physical exercise, diet, and smoking and alcohol drinking status. The simple food-frequency questionnaire (FFQ25) was used to evaluate dietary patterns.

This study conformed to the ethical principles stated in the Declaration of Helsinki, and the protocol was approved by affiliated LiYuan Hospital of Tongji Medical College, Huazhong University of Science and Technology (permission no. [2020] IEC (A016)). All participants or close relatives or caregivers of patients with cognitive impairment provided informed consent.

### Collection of Blood and Fecal Samples

All participants were invited for a blood draw in the morning after an 8-h overnight fasting. Blood samples were centrifuged at 3,000×*g* for 15 min at 4°C after 30 min of clotting at room temperature. A blood cell analyzer was applied to examine complete blood count, and an automatic biochemical analyzer was used to assess C-reactive protein (CRP), blood glucose, blood lipids, liver, and renal function. The serum fraction was collected in 0.3 ml aliquots and stored at −80°C. The serum levels of IL-6, HMGB1, and zonulin were measured using ELISA with a detection range of 5–400 pg/ml, 10–800 ng/ml, and 3–240 ng/ml, respectively (BioSwamp, China). All participants were guided to obtain a standardized collection of fecal samples. Fresh fecal samples (about 1–3 g) were collected at home in a dry aseptic exclusive stool collector, and samples were immediately stored at −20°C. Collected fecal samples were sent to the laboratory within 2 h and stored at −80°C.

### DNA Extraction and Microbiome Profiling

Genomic DNA was extracted from fecal samples using the CTAB/SDS method, and the V3–V4 region of the 16S rRNA gene was amplified. The forward primer used was 341F (5′-CCTAYGGGRBGCASCAG-3′), and the reverse primer was 806R (5′-GGACTACHVGGGTWTCTAAT-3′). A reaction containing 15 μl of Phusion^®^ High-Fidelity PCR Master Mix (New England Biolabs), 0.2 μM of forward and reverse primers, and 10 ng template DNA in 30 μl total volume was used. The PCR reactions were carried out and the PCR products were extracted *via* electrophoresis using 2% agarose gels and purified using the GeneJET Gel Extraction Kit (Thermo Scientific). Samples with a single amplification product were selected for further analysis. After DNA library preparation, sequencing was performed on an Illumina NovaSeq platform at Novogene Company (Beijing, China). We transmitted the raw sequence data to the National Center for Biotechnology Information (NCBI) database, and the accession number was PRJNA704230.

### Analysis of 16S Amplicon Sequencing Data

Fastq data were screened and filtered using a maximum false rate of 1% as evaluation standard using VSEARCH software v1.1. All replicative (repeated) data were identified and removed. The data were further denoised and clustered into operational taxonomic units (OTUs) with a 97% threshold of distance-based similarity using USEARCH software. After identifying and deleting chimeras, the OTU-profiling tables were constructed, and the taxonomy of gene sequences was assigned using the RDP database. QIIME version 2 was used to conduct alpha/beta diversity analyses. Alpha diversity was calculated using the Shannon diversity index, Faith’s phylogenetic distance index, observed OTUs, and Pielou’s evenness index, referring to richness and uniformity of the gut microbiota within the sample. Beta diversity was estimated using the weighted UniFrac and Bray–Curtis to calculate the distances between the samples and visualized by principal coordinate analysis (PCoA). The statistical differences in beta diversity were determined using permutational multivariate analysis of variance (PERMANOVA) test, as implanted in QIIME2 with the default of 999 permutations. Analysis of similarities (ANOSIM) was conducted based on Bray–Curtis distance. Linear discriminant analysis (LDA) of the effect size (LEfSe) was used to identify the significantly differential taxa between the two groups. The threshold of LDA scores was set to 3.5, and the *α* value was set at 0.01. Receiver operating characteristic (ROC) curve analysis was used to evaluate the clinical diagnostic value of the ability of the microbial markers to differentiate between older adults with frailty and healthy controls.

### Statistical Analysis

SPSS (ver. 26.0, SPSS Inc., Chicago, IL) and R (ver. 4.0.3, R Foundation for Statistical Computing) software were used to process the data. The measurement data were expressed as the mean with standard deviation (*x* ± *s*) for statistical description and analyzed with *t*-tests. The numerical data were expressed as percentage, and the comparison of differences was assessed with the *χ* ^2^ test. The dietary patterns were extracted by factor analysis, and the groups were divided according to the maximum score of dietary patterns. Correlation analysis of the top 100 most abundant taxa in two groups was performed using the Pearson correlation coefficient. The results with Spearman’s absolute value greater than 0.6 and *P*-value less than 0.05 were retained. Correlations between fecal microbiota with distinct differences and serum markers were calculated using Spearman’s rank correlation with the ‘‘cor. test” package in R. A *P*-value of <0.05 was considered statistically significant. We used random forest (RF) models to predict the occurrence of frailty using the “RandomForest” package in R.

## Results

### Participant Characteristics

Between July 2020 and October 2020, 47 older adults with frailty and 47 healthy controls matched for gender and age were included in this cross-sectional study. Participants had a mean age of 80.72 ( ± 5.75) years, ranging from 70 to 92 years. The demographic characteristics and clinical parameters of the participants are summarized in **Table 1**. The age and sex ratio were not statistically significantly different between the two groups. The body mass index (BMI) and grip strength, as parameters of frailty, were lower in the frail group. Older adults with frailty had lower Mini Nutritional Assessment-Short Form (MNA-SF), Mini-Mental State Examination (MMSE), and Geriatric Depression Scale (GDS) scores than healthy controls. The number of participants with a college education or above was higher in the control group compared with the frail group, having a significant difference. Older adults with frailty had a higher incidence of comorbidity and were more likely to take more medicine. Hypertension was the most common co-occurring disease, followed by coronary artery disease, diabetes mellitus, and cerebral ischemic stroke. The incidence of smoking and drinking at present was higher in healthy controls. No apparent differences were found in the levels of fasting blood glucose, creatinine, and AST, but frail participants had lower levels of ALT, total cholesterol, low-density lipoprotein, and triglycerides. These results are summarized in **Table S1**. The daily dietary intake in older adults with frailty and healthy controls is shown in **Table S2**. Four dietary patterns (bean products and nuts, rice and tubers, red meat and wine, coarse grains) were identified according to the results of the FFQ25. These dietary patterns, except for dietary pattern rice and tubers, were not significantly different in the two groups.

**Table 1:** Baseline characteristics of the study sample.

Features	Total (*n* = 94)	Frailty (*n* = 47)	Control (*n* = 47)	*P*-value
Age (years)	80.72 ± 5.75	81.68 ± 6.15	79.76 ± 5.22	0.107
Gender (male, %)	44, 46.8%	23, 48.94%	21, 44.68%	0.679
BMI (kg/m^2^)	23.27 ± 2.89	21.85 ± 2.38	24.02 ± 3.18	0.011*
College education or above, *n* (%)	53 (56.38%)	21 (44.68%)	32 (68.09%)	0.038*
Living with partner, *n* (%)	30 (31.91%)	12 (25.53%)	18 (38.30%)	0.269
Drug types, *n* (%)	4.50 ± 2.67	6.62 ± 1.98	2.38 ± 1.13	<0.001*
Grip strength (kg)	21.12 ± 8.88	13.79 ± 3.02	28.46 ± 6.39	<0.001*
MNA-SF scores	9.30 ± 3.49	6.56 ± 1.56	12.53 ± 1.01	<0.001*
MMSE scores	23.48 ± 7.64	19.04 ± 8.63	27.91 ± 1.62	<0.001*
GDS scores	12.07 ± 8.85	19.08 ± 7.44	5.06 ± 1.73	<0.001*
Comorbidities				
HTN, *n* (%)	56 (59.57%)	29 (61.70%)	27 (57.44%)	0.834
CAD, *n* (%)	40 (42.55%)	28 (59.57%)	12 (25.53%)	0.002*
DM, *n* (%)	24 (25.53%)	15 (31.91%)	9 (19.15%)	0.237
CI, *n* (%)	20 (21.28%)	17 (36.17%)	3 (6.38%)	<0.001*
AD, *n* (%)	9 (9.57%)	9 (19.15%)	0 (0.00%)	0.005*
CHF, *n* (%)	7 (7.45%)	7 (14.89%)	0 (0.00%)	0.018*
Lifestyle				
Smoking at present, *n* (%)	14 (14.89%)	3 (6.38%)	11 (23.40%)	0.043*
Drinking at present, *n* (%)	16 (17.02%)	2 (4.25%)	14 (29.79%)	0.003*
Dietary pattern				
Bean products and nuts	25 (26.60%)	11 (44.00%)	14 (56.00%)	0.484
Rice and tubers	22 (23.40%)	16 (72.72%)	6 (27.27%)	0.015*
Red meat and wine	23 (24.75%)	10 (43.48%)	13 (56.52%)	0.472
Coarse grains	24 (25.53%)	10 (41.67%)	14 (58.33%)	0.344

Data are shown as the mean ± SD or n (%). P-value was expressed as two-dependent t-test or chi-squared test.

BMI, body mass index; MNA-SF, Mini Nutritional Assessment-Short Form; MMSE, Mini-Mental State Examination; GDS, Geriatric Depression Scale; HTN, hypertension; CAD, coronary artery disease; DM, diabetes mellitus; CI, cerebral ischemic stroke; AD, Alzheimer’s disease; CHF, chronic heart failure.

*P < 0.05.

### Increased Levels of Inflammatory and Intestinal Permeability Markers in Older Adults With Frailty

**Figure 1:**
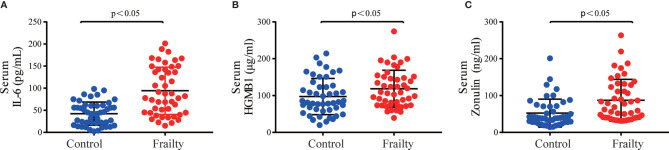
Serum inflammatory cytokine concentrations of **(A)** IL-6 and **(B)** HMGB1 and intestinal permeability biomarker **(C)** zonulin among older adults with frailty and healthy controls. *P < 0.05 for differences between groups.

There was no statistically significant difference in the total number of white blood cells and neutrophils between the two groups, but frail participants had lower lymphocyte levels and higher CRP levels. The result is shown in **Table S1**. The serum inflammatory biomarkers IL-6 and HGMB1 and the intestinal permeability marker zonulin-1 were higher among older adults with frailty than in healthy adults (*P* < 0.05 for all, **Figure 1**).

### Alpha and Beta Diversity Between Older Adults With Frailty and Healthy Controls

**Figure 2:**
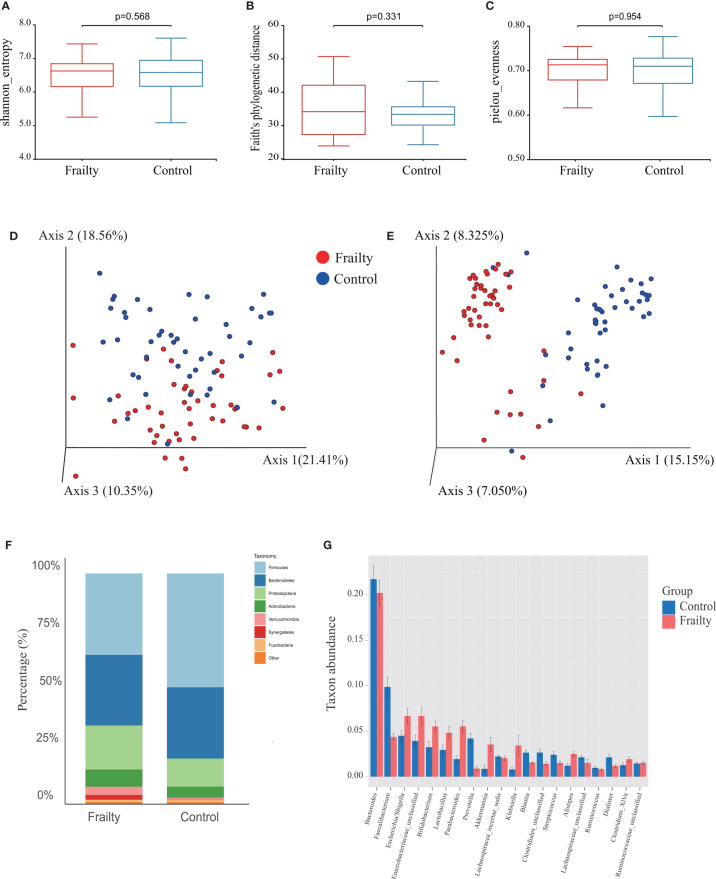
Fecal microbiota alpha and beta diversity indices in older adults with frailty and healthy controls. The box plots depict differences in microbiome diversity indices between frail and control groups using the **(A)** Shannon diversity index, **(B)** Faith’s phylogenetic distance index, and **(C)** Pielou’s evenness index. Each box plot represents the median, interquartile range, and minimum and maximum values. The level of similarity between the microbial communities detected in frail (red) and control (blue) groups was assessed *via* principal coordinate analysis (PCoA) based on **(D)** weighted UniFrac and **(E)** Bray–Curtis distances. These analyses revealed the **(F)** top 8 most abundant phyla and the **(G)** top 20 most abundant genera between the frail and control groups.

The rarefaction curves of all samples reached plateaus, which suggested that sequencing depth was enough for the following analyses (**Figure S1A**). Analysis of the collected fecal samples showed that no statistical differences were observed in alpha diversity using the Shannon diversity index (**Figure 2A**, *P* = 0.568), Faith’s phylogenetic distance index (**Figure 2B**, *P* = 0.331), observed OTUs (*P* = 0.245), and Pielou’s evenness index (**Figure 2C**, *P* = 0.954), suggesting that the richness and uniformity of the gut microbiota in the two groups had no significant differences. Beta diversity is used to assess the variability in fecal microbiota structures and was characterized for samples using weighted UniFrac (**Figure 2D**) and Bray–Curtis distances (**Figure 2E**). Most samples showed distinct clustering between the frail and control groups in PCoA analysis. ANOSIM of Bray–Curtis distances showed that this clustering was significant (Bray–Curtis: *R* = 0.361, *P* = 0.001).

### Alteration in the Taxa Between Older Adults With Frailty and Healthy Controls

We further analyzed the specific changes of gut microbiota in frail participants. At the phylum level, *Firmicutes*, *Bacteroidetes*, *Proteobacteria*, *Actinobacteria*, *Verrucomicrobia*, *Synergistetes*, and *Fusobacteria* were identified as the most abundant sequences (**Figure 2F**). Firmicutes were significantly decreased, whereas *Proteobacteria*, *Actinobacteria*, *Verrucomicrobia*, and *Synergistetes* were overrepresented in the frail group (**Figure S1C**, both *P* < 0.05). At the genus level, the top 20 most abundant bacterial taxa are shown in **Figure S1D**. In comparison with healthy controls, the relative abundances of *Faecalibacterium*, *Prevotella*, *Blautia*, and *Streptococcus*, and of an unknown genus in the family of *Clostridiales*, were significantly decreased in the frail group. However, the levels of taxa *Escherichia/Shigella*, *Bifidobacterium*, *Lactobacillus*, *Parabacteroides*, *Akkermansia*, *Klebsiella*, and *Alistipes*, and of an unknown genus in the family of *Enterobacteriaceae*, were increased (**Figure 2G**).

**Figure 3:**
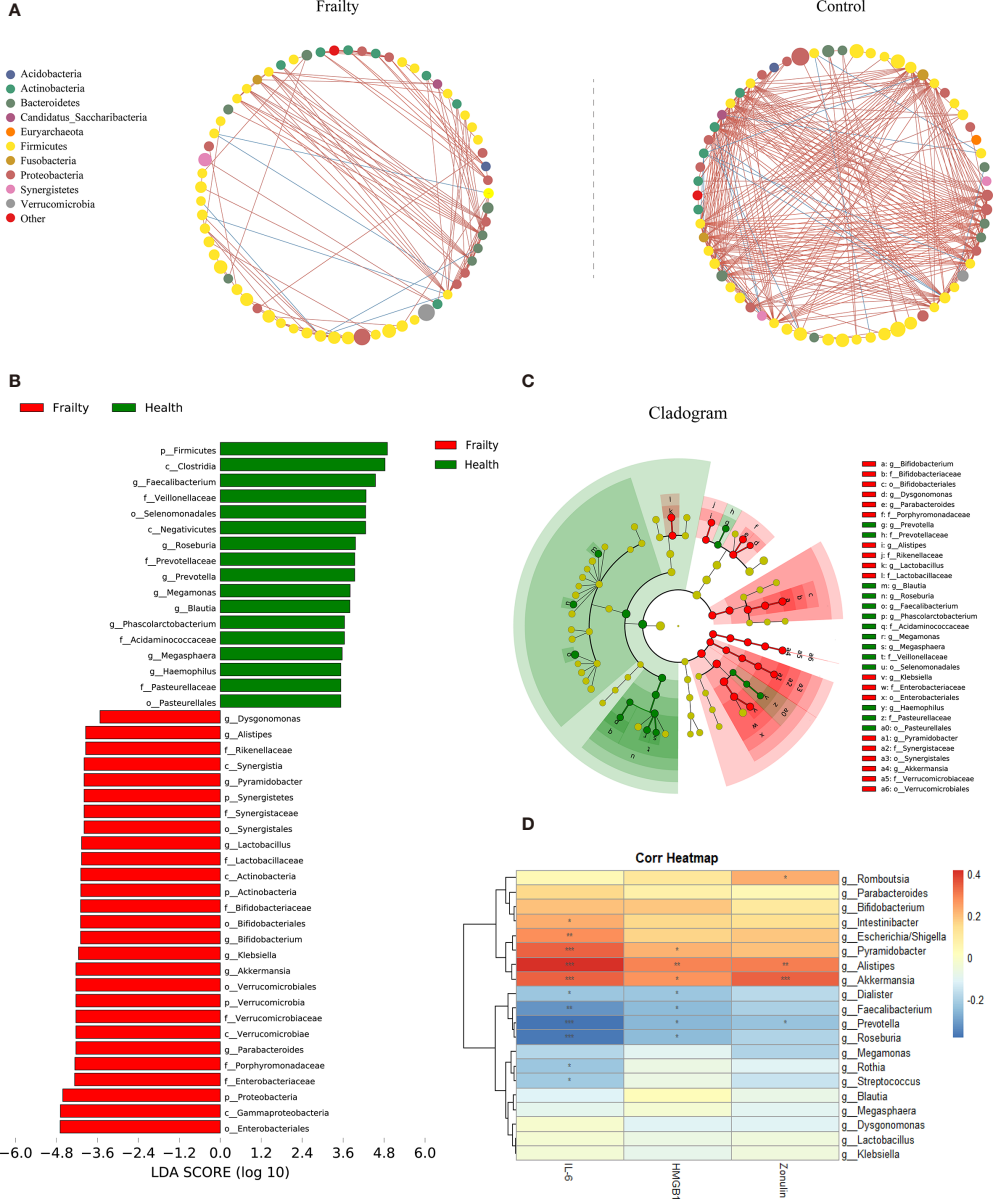
The distinctive gut microbial co-abundance networks between frail and control group **(A)**. The relationships among the top 100 most abundant taxa in the frail and control groups were evaluated using Spearman’s correlation analysis. The size of the nodes was proportional to the relative abundance. The positive and negative correlations were represented using red and blue lines, respectively. Bacterial taxa differentially abundant exhibited by older adults with frailty and healthy controls. **(B)** A linear discriminant analysis [LDA; (log10) > 3.5] and **(C)** effect size (LEfSe) analysis revealed significant differences (*P* < 0.01) in the microbiota exhibited by the frail (red, positive score) and control (green, negative score) groups. **(D)** Heatmaps showing correlations between the top 20 differentially abundant microbiota genera, inflammatory cytokines IL-6 and HMGB1, and the intestinal permeability marker zonulin-1. *P < 0.1, **P < 0.05, ***P < 0.01.

The top 100 most abundant genera were further evaluated using Spearman’s correlation analysis; 64 genera could interact with each other in the frailty group and 65 genera in the control group. The relation network pictures were constructed, respectively (**Figure 3A**). Spearman’s correlation analysis showed that the intergenus interactions were more common in healthy controls than older adults with frailty. The red line revealed that Firmicutes members were more likely to form a positive co-occurrence relationship in healthy controls than in older adults with frailty.

The LDA and LEfSe analyses were used to identify the gut microbiota differences between the two groups. There were significant differences in the fecal microbiota collected from older adults with frailty and healthy controls. The relative abundances of *Parabacteroides*, *Akkermansia*, *Klebsiella*, *Bifidobacterium*, *Lactobacillus*, *Pyramidobacter*, *Alistipes*, and *Dysgonomonas* were found to be higher in older adults with frailty. In contrast, *Faecalibacterium*, *Roseburia*, *Prevotella*, *Megamonas*, *Blautia*, *Phascolarctobacterium*, *Megasphaera*, and *Haemophilus* were found to be enriched in the healthy controls compared with older adults with frailty (**Figures 3B, C**).

### Associations Among Gut Microbiota and Clinical Parameters

We analyzed the potential relevance of fecal microbiota with distinct differences in abundance and the inflammatory factors IL-6 and HMGB1 and the intestinal permeability biomarker zonulin. The results are shown in **Figure 3D**. The predominant genera in the frail group, *Escherichia/Shigella*, *Pyramidobacter*, *Alistipes*, and *Akkermansia*, were positively correlated with IL-6, while *Faecalibacterium*, *Prevotella*, and *Roseburia*, the predominant genera in the control group, were negatively correlated with IL-6. *Alistipes* were found to be positively correlated with HGMB1. Both *Alistipes* and *Akkermansia* were positively correlated with zonulin.

### Gut Microbiota Composition Can Be Used to Distinguish Older Adults With Frailty From Healthy Controls

**Figure 4:**
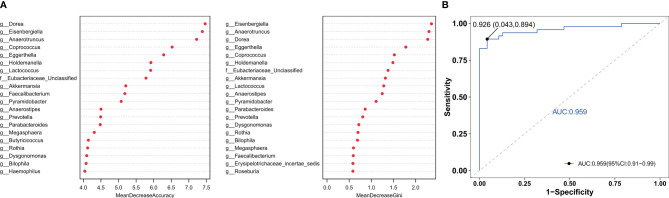
The RF model was used to build a predictive model of genus-level abundance taxa **(A)**. The relative importance of each genus in the predictive model was evaluated using the mean decreasing accuracy and the Gini coefficient. **(B)** Receiver operating characteristic curve (ROC) generated using 20 genera in the fecal microbiota.

To find the fecal microbiota for sample classification, we applied RF to build a predictive model. The mean decreasing accuracy and the Gini coefficient for fecal microbiota were used to evaluate the relative importance of each genus in the predictive model. The top 20 key microbiota at the genus level predictive of frailty are shown in **Figure 4A**. We used ROC analysis to evaluate the accuracy of the sample classifications (**Figure 4B**). In the specificity and sensitivity analysis, the area under the curve (AUC) was 95.9%.

## Discussion

Soysal et al. analyzed the relationship between inflammation and frailty through a systematic review and meta-analysis and concluded that frailty and prefrailty are associated with higher inflammatory parameters, especially CRP and IL-6. This is in agreement with the results of our studies. The reason for frailty-related inflammation is not clear. Previous studies have shown that age-associated microbial dysbiosis can lead to aging-related inflammation and early mortality in mice. The process may involve increasing intestinal permeability and translocation of bacterial components, further causing a state of immune activation, systemic inflammation, and damage of cellular antibacterial function (Thevaranjan et al., 2017). The zonulin family is a group of proteins that correlates with increased epithelial permeability by regulating intercellular tight junctions (Barbaro et al., 2020). In this study, we show that the levels of serum inflammatory cytokines and intestinal permeability of community-dwelling older adults with frailty are higher than those of healthy controls.

A previous report investigated the gut microbiota changes associated with frailty in a younger community-dwelling population and found a negative correlation between early frailty and gut microbiota diversity (Jackson et al., 2016). Higher diversity is generally considered to be related to health, and reduced diversity is associated with health issues such as metabolic disease (Lim et al., 2017), Alzheimer’s disease (Calvani et al., 2018), and inflammatory bowel disease (Matsuoka and Kanai, 2015; Zuo et al., 2018). Our analysis suggests that the richness and uniformity of the gut microbiota in the two groups are not significantly different. Age- and gender-matched older adults were selected as controls in our study. Many observational studies have demonstrated that gut microbiota changes consist of reduced diversity and overexpression of pathobionts as people age (Kim and Jazwinski, 2018; Calvani et al., 2018). Aging-related diseases, including frailty, are based on the degeneration of body organs, and the gut microbiota is thought of as a forgotten organ in human health and disease. Physical frailty and sarcopenia (PF&S) show remarkable clinical overlap and share multisystem disorders (Wilson et al., 2017). No significant differences in alpha diversity between PF&S and age- and gender-matched non-PF&S older adults were noted, and these results are in line with our study (Picca et al., 2019).

The commensal genera such as *Faecalibacterium*, *Prevotella*, *Roseburia*, *Megamonas*, and *Blautia* were decreased in the frail group. *Faecalibacterium prausnitzii*, the only species of the genus *Faecalibacterium*, is a highly abundant gut microbiota with anti-inflammatory and immunoregulatory properties in healthy human individuals (Laursen et al., 2017). It is considered to be a marker of gut health. *Prevotella* is a dominant genus member of the Bacteroidetes and is more widespread in individuals with non-Western lifestyles (Ley, 2016; Tett et al., 2019). The role of *Prevotella* to the body has remained controversial and might be dependent on diet. Previous studies have shown that improved glucose metabolism and insulin tolerance induced by dietary fiber is associated with an increased abundance of Prevotella (Kovatcheva-Datchary et al., 2015). *Roseburia* is an anaerobic bacterium belonging to the *Clostridium cluster XIVa* (Seo et al., 2020), which has been shown to play a major role in maintaining the intestinal barrier function and immune defense (Kasahara et al., 2018; Seo et al., 2020). A reduced abundance of SCFA-producing bacteria *Megamonas* was also found in heart failure (Hayashi et al., 2018), depression (Cheung et al., 2019), multiple system atrophy (Wan et al., 2019), and Bechet’s disease (Shimizu et al., 2016). However, little research on the relevance of *Megamonas* in human health has been conducted. In addition, *Blautia* in the gut microbiota of obese children might help reduce intestinal inflammation (Benitez-Paez et al., 2020).

The frail group was significantly enriched for *Parabacteroides* and *Klebsiella*. *Parabacteroides distasonis* is one of the core bacteria in the human body. A correlation analysis showed that its content was negatively correlated with obesity, nonalcoholic fatty liver disease, diabetes, and other diseases, suggesting that it may play a positive regulatory role in glucose and lipid metabolism (Wang et al., 2019). Weight loss is a sign of frailty syndrome. Cancer cachexia is an extremely frail state involving multiple organs, including weight loss, muscle consumption, and systemic inflammation. Cachectic mice exhibit an increase in *Enterobacteriaceae* and *Parabacteroides* (Herremans et al., 2019). Pglyrp-regulated gut microflora *P. distasonis* can enhance DSS-induced colitis in mice, identifying *P. distasonis* as colitis-promoting species (Dziarski et al., 2016). Higher levels of stress are associated with increased Crohn’s disease activity. The relative abundances of Parabacteroides are significantly higher in patients with high perceived stress scale (PSS) scores compared with those with low PSS scores. However, whether the abundance variation of *Parabacteroides* is the cause or a consequence of increased intestinal inflammation remains to be further studied (Mackner et al., 2020). Nakamoto et al. identified *Klebsiella pneumoniae* in the gut microbiota of patients with primary sclerosing cholangitis that can disrupt the epithelial barrier to initiate bacterial translocation and promote liver inflammatory responses (Nakamoto et al., 2019). Oral pathobionts *Klebsiella* can also promote colitis by the colonization of the gut and activation of the inflammasome in colonic mononuclear phagocytes (Kitamoto et al., 2020).

Additionally, a higher level of the bacteria *Akkermansia* was found in the frail group. *Akkermansia* is a symbiotic bacterium colonizing the mucosal layer that uses mucin as carbon and nitrogen element sources and has been widely disputed in recent years (Zhang et al., 2019). *Akkermansia muciniphila* is one of the top 20 most abundant species detected in the human gut microbiota (Arumugam et al., 2011; Zhang et al., 2019). Its colonization starts in early life, but the concentration of *A. muciniphila* in fecal samples was decreased in older adults aged 80 to 82 (Collado et al., 2007). Many researchers also revealed a higher abundance of *Akkermansia* in centenarians aged 99 to 104 and in semi-supercentenarians aged 105 to 109 (Kong et al., 2016). However, the rate of frailty increases with age, and we should be more concerned about the beneficial effect of microbiota composition in nonfrailty older adults instead of in all older adults. Previous studies have shown that a low-fiber diet promotes the activity and expansion of colonic mucus-degrading bacteria *A. muciniphila*, as well as increases in the activity of mucus-targeting enzymes, which can damage the intestinal mucosal barrier function and enhance pathogen susceptibility (Desai et al., 2016). Considering that aging is accompanied by multiple organ functional aging, we suspect that a low abundance of *Akkermansia* may be more conducive to healthy aging. *Alistipes* are pathogenic in colitis and colorectal cancer (Moschen et al., 2016; Parker et al., 2020) and are related to liver fibrosis (Rau et al., 2018) and signs of depression (Bangsgaard Bendtsen et al., 2012). In hypertension, the increase in *Alistipes finegoldii* was also observed to be a potential driver for gut barrier dysfunction and inflammation (Kim et al., 2018).

Although our study has very strict inclusion and exclusion criteria to control confounding factors, we cannot completely exclude the possibility that some factors such as comorbidity and lifestyle including smoking and drinking may affect our results, which is a common difficulty in the study of gut microbiota. It should be noted that comorbidity was widespread among older adults with frailty and has been considered synonymous with frailty (Fried et al., 2001). However, many frailty-related diseases such as hypertension and diabetes have been reported to be associated with gut microbiota (Breuninger et al., 2021). We recorded the variables of drinking and smoking at present in the healthy control group and frail group. However, the amount of smoking and drinking and smoking cessation were not recorded. Some researchers have proposed that gut microbial biodiversity was higher in human drinkers than nondrinkers (Kosnicki et al., 2019). Smoking and smoking cessation led to changes in the gut microbiota (Sublette et al., 2020). Therefore, it is not clear how these factors affect our results, which is one of our main limitations. This problem needs to be clarified by matching these variables between the groups being compared.

There are some other general limitations to our study. Firstly, our work is based on a small sampling of older adults living in a community. Thus, the generalization of these findings must be carefully considered, and studies in different populations worldwide are required to confirm these data. Secondly, this study was cross-sectional based on community, and a longitudinal study might provide more robust evidence. Thirdly, fecal microbiota may not provide or reflect the whole bowel microbial environment, and the stool consistency, which is strongly associated with gut microbiota richness and composition, was not recorded in this study. Fourthly, the 16S rRNA gene sequencing method exhibits limited efficacy, as it cannot provide direct data of gene function and more in-depth analysis of species. Analyses of gut microbiota changes using metagenomics sequencing are therefore required. Due to the limited sample size, the predictive power of 20 genera random forests has not been evaluated. Fifthly, some gastrointestinal symptoms such as abdominal pain, constipation, and diarrhea that may be related to gut microbiota composition were not recorded in this study.

## Conclusions

In conclusion, the results of the present study indicate that the inflammatory cytokines IL-6 and HGMB1 and the intestinal permeability biomarker zonulin were significantly increased in older adults with frailty. We provide a detailed comparison of gut microbiota in both frail participants and healthy controls using high-throughput sequencing and found that the bacterial community composition was significantly different from that of healthy controls. Some of the gut microbiota have a close association with inflammatory and intestinal mucosal permeability biomarkers. Focusing on a target for the treatment methods based on gut microbiota adjustment may represent an effective therapy for preventing and managing frailty. To develop more reliable microbiome biomarkers of frailty, large-scale prospective studies and rigorous animal experiments should be conducted.

## Data Availability Statement

The datasets presented in this study can be found in online repositories. The names of the repository/repositories and accession number(s) can be found below: (https://www.ncbi.nlm.nih.gov/), PRJNA704230.

## Ethics Statement

The studies involving human participants were reviewed and approved by Affiliated LiYuan Hospital of Tongji Medical College, Huazhong University of Science and Technology. The patients/participants provided their written informed consent to participate in this study.

## Author Contributions

Clinical analyses and manuscript writing: YX and DC. Sample collection and DNA extraction: HL, YD, ZH, HY, and JH. Statistical analyses: YW and YX. Sequencing analyses and management: MW and XJ. Project supervision and manuscript revision: ZX. Study design, project management, financial support, and manuscript revision: ZX. All authors contributed to the article and approved the submitted version.

## Funding

This study was supported by the National Key Research and Development Program of China (2018YFC2002000).

## Conflict of Interest

The authors declare that the research was conducted in the absence of any commercial or financial relationships that could be construed as a potential conflict of interest.

## Publisher’s Note

All claims expressed in this article are solely those of the authors and do not necessarily represent those of their affiliated organizations, or those of the publisher, the editors and the reviewers. Any product that may be evaluated in this article, or claim that may be made by its manufacturer, is not guaranteed or endorsed by the publisher.

## Supplementary Material

Supplementary Figure 1 Rarefaction curve: the abscissa represents the number of randomly selected sequences, and the ordinate represents the number of OTUs **(A)**. Each curve represents a sample, all rarefaction curves reached plateaus, suggesting accurate sequencing depth. The level of similarity between the fecal microbial communities detected in the frail and control groups was assessed using an unweighted Analysis of similarity (ANOSIMs) **(B)**. These analyses revealed the **(C)** top 8 most abundant phyla and the **(D)** top 20 most abundant genera between the frail and control groups.
