# Supplementary material for: Altered Fecal Microbiota Composition in Older Adults With Frailty
Source: Front Cell Infect Microbiol. 2021 Aug 17;11:696186. doi: 10.3389/fcimb.2021.696186 (PMC8415883; doi:10.3389/fcimb.2021.696186)
Supplement: Supplementary file 2 [file Table_1.doc]

| Biomarker | Frailty  (n=47) | Control  (n=47) | P-value |
| --- | --- | --- | --- |
| Blood routine examination | | | |
| White blood cells (x10^9^/L) | 5.33±1.31 | 5.78±1.08 | 0.076 |
| Red blood cell (x10^9^/L) | 3.67±0.59 | 4.64±0.37 | ＜0.001* |
| Neutrophils (x10^9^/L) | 3.56±1.57 | 3.38±0.86 | 0.486 |
| Lymphocytes (x10^9^/L) | 1.30±0.53 | 2.07±1.02 | ＜0.001* |
| Platelet (x10^9^/L) | 183.66±58.28 | 215.49±51.48 | 0.006* |
| Blood biochemical index | | | |
| Fasting blood glucose (mmol/L) | 6.90±3.70 | 6.05±1.99 | 0.172 |
| Total cholesterol (mmol/L) | 3.84±0.91 | 4.86±1.13 | ＜0.001* |
| Low-density lipoprotein (mmol/L) | 2.16±0.80 | 2.83±0.97 | ＜0.001* |
| Triglycerides (mmol/L) | 1.19±0.67 | 1.74±1.24 | 0.008* |
| ALT (U/L) | 15.19±11.46 | 21.50±12.10 | 0.011* |
| AST (U/L) | 21.58±10.54 | 25.99±14.40 | 0.093 |
| Creatinine (μmoI/L) | 78.25±59.54 | 79.74±14.50 | 0.867 |
| Inflammatory indicators | | | |
| hs-CRP (mg/L) | 11.15±14.45 | 1.94±2.02 | ＜0.001* |
| IL-6 (pg/mL)) | 121.89±53.02 | 59.49±25.94 | ＜0.001* |
| HGMB1 (ng/mL) | 118.31±49.69 | 97.38±48.78 | 0.044* |
| Intestinal permeability biomarker | | | |
| Zonulin (ng/mL) | 87.29±56.24 | 52.47±37.59 | ＜0.001* |

**Table S1** The clinical biomarkers levels exhibited by older adults with frailty and healthy controls.

Data are shown as the mean±SD.

1. value was expressed as two-dependent t test.

*p < 0.05
