# Supplementary material for: Altered Fecal Microbiota Composition in Older Adults With Frailty
Source: Front Cell Infect Microbiol. 2021 Aug 17;11:696186. doi: 10.3389/fcimb.2021.696186 (PMC8415883; doi:10.3389/fcimb.2021.696186)
Supplement: Supplementary file 3 [file Table_2.doc]

**TableS2** The daily dietary intake in older adults with frailty and healthy controls.

| Food proportion | Robust  M (P25, P75) | Frailty  M (P25, P75) | Z | P |
| --- | --- | --- | --- | --- |
| Rice (g/d) | 100(100~150) | 50(50~150) | -4.851 | 0.000* |
| Porridge (g/d) | 150(100~150) | 150(100~150) | -0.393 | 0.694 |
| Flour (g/d) | 60(60~60) | 60(30~60) | -1.961 | 0.050 |
| Desserts (g/d) | 10(5~15) | 10(0~15) | -0.524 | 0.600 |
| Fried (g/d) | 0(0~20) | 0(0~0) | -0.748 | 0.454 |
| Stuffings (g/d) | 0(0~30) | 0(0~30) | -1.064 | 0.287 |
| Coarse food (g/d) | 50(30~60) | 50(30~50) | -4.530 | 0.000* |
| Tubers (g/d) | 60(30~60) | 30(0~30) | -4.857 | 0.000* |
| Milk (g/d) | 150(150~200) | 100(100~150) | -2.464 | 0.014* |
| Eggs (g/d) | 60(60~60) | 60(60~60) | -1.149 | 0.251 |
| Red meat (g/d) | 30(30~50) | 50(30~60) | -2.432 | 0.015* |
| Poultry (g/d) | 25(20~30) | 20(20~30) | -1.753 | 0.080 |
| Processed meat (g/d) | 6(4~6) | 6(5~8) | -2.719 | 0.007* |
| Aquatic products (g/d) | 30(0~30) | 30(0~30) | -2.513 | 0.012* |
| Seafood (g/d) | 0(0~30) | 0(0~30) | -1.839 | 0.066 |
| Bean Products (g/d) | 30(0~60) | 0(0~30) | -2.642 | 0.008* |
| Nuts (g/d) | 20(0~30) | 0(0~30) | -0.184 | 0.236 |
| Dark color vegetables (g/d) | 150(100~150) | 100(80~100) | -6.484 | 0.000* |
| Light-color vegetable (g/d) | 70(50~80) | 50(30~60) | -4.953 | 0.000* |
| Mushrooms (g/d) | 20(20~30) | 20(10~30) | -0.442 | 0.658 |
| Fruits (g/d) | 100(100~150) | 60(0~100) | -3.863 | 0.000* |
| Sweetened drinks (g/d) | 0(0~0) | 0(0~0) | -2.285 | 0.022* |
| Beer (g/d) | 0(0~100) | 0(0~0) | -3.805 | 0.000* |
| Rice wine (g/d) | 0(0~0) | 0(0~0) | -0.200 | 0.841 |
| White liquor (g/d) | 0(0~0) | 0(0~0) | -2.517 | 0.012* |

Data are shown as the the median (quartile) [M (P25, P75)]

The data applies non-parametric test.

*p < 0.05
